# The pacing differences in performance levels of marathon and half-marathon runners

**DOI:** 10.3389/fpsyg.2023.1273451

**Published:** 2023-12-21

**Authors:** Ljubica Ristanović, Ivan Cuk, Elias Villiger, Stanimir Stojiljković, Pantelis T. Nikolaidis, Katja Weiss, Beat Knechtle

**Affiliations:** ^1^Faculty of Sport and Physical Education, University of Belgrade, Belgrade, Serbia; ^2^Klinik für Allgemeine Innere Medizin, Kantonsspital St. Gallen, St. Gallen, Switzerland; ^3^Exercise Physiology Laboratory, Nikaia, Greece; ^4^School of Health and Caring Sciences, University of West Attica, Athens, Greece; ^5^Institute of Primary Care, University of Zurich, Zurich, Switzerland; ^6^Medbase St. Gallen Am Vadianplatz, St. Gallen, Switzerland

**Keywords:** endurance, long-distance races, running, speed, strategy, variability

## Abstract

**Introduction:**

Many studies indicate a considerable impact of optimal pacing on long-distance running performance. Given that the amount of carbohydrates in metabolic processes increases supralinearly with the running intensity, we may observe differences between the pacing strategies of two long-distance races and different performance levels of runners. Accordingly, the present study aimed to examine the differences in pacing strategies between marathon and half-marathon races regarding the performance levels of runners.

**Methods:**

The official results and split times from a total of 208,760 (marathon, *N* = 75,492; half-marathon, *N* = 133,268) finishers in the “Vienna City Marathon” between 2006 and 2018 were analyzed. The percentage of the average change of speed for each of the five segments (CS 1–5), as well as the absolute change of speed (ACS) were calculated. The CS 1–5 for the marathon are as follows: up to the 10th km, 10th – 20th km, 20th – 30th km, 30th – 40th km, and from the 40th km to the 42.195 km. For the half-marathon, the CS 1–5 are half of the marathon values. Four performance groups were created as quartiles of placement separately for sexes and races: high-level (HL), moderate to high-level (MHL), moderate to low-level (MLL), and low-level (LL).

**Results:**

Positive pacing strategies (i.e., decrease of speed) were observed in all performance groups of both sex and race. Across CS 1–5, significant main effects (*p* < 0.001) were observed for the segment, performance level, and their interaction in both sex and race groups. All LL groups demonstrated higher ACS (men 7.9 and 6.05%, as well as women 5.83 and 5.49%, in marathon and half-marathon, respectively), while the HL performance group showed significantly lower ACS (men 4.14 and 2.97%, as well as women 3.16 and 2.77%, in marathon and half-marathon, respectively). Significant main effects (*p* < 0.001) for the race were observed but with a low effect size in women (ŋ^2^ = 0.001).

**Discussion:**

Better runners showed more even pacing than slower runners. The half-marathoners showed more even pacing than the marathoners across all performance groups but with a trivial practical significance in women.

## 1 Introduction

Pacing is often referred to as the regulation of the distribution of energy expenditure during human activities, especially in a sport setting, to achieve the best result (Tucker and Noakes, 2009). Many studies indicate a significant impact of optimal pacing on long-distance running performance (Skorski and Abbiss, 2017; De Leeuw et al., 2018; Kais et al., 2019; Foster et al., 2023). Furthermore, an optimal pacing strategy during long-distance running races plays a vital role in preventing homeostatic disorders (Tucker and Noakes, 2009; de Koning et al., 2011; Foster et al., 2023), thus making the race more enjoyable for runners (Cuk et al., 2019a,b), while decreasing the risk of musculoskeletal injuries (de Koning et al., 2011).

An optimal pacing strategy depends on a race’s duration, the consequences of the speed deceleration with the loss of power output (Foster et al., 2023), the experience of the athlete, and the physiological capacity of each of them (St Clair Gibson et al., 2006). However, a complex balancing system between optimal performance and sustainability of homeostasis is the main requirement of the pacing strategy during activity (Abbiss and Laursen, 2008; Skorski and Abbiss, 2017; Foster et al., 2023). Consequently, an even pace might be the most metabolically efficient strategy for finishing a long-distance race at a given time (Rapoport, 2010). In particular, the critical parameter is the effort level (% VO_2_max), which on a steady track is approximately proportional to the runner’s pace (Rapoport, 2010).

In addition to the influence of sex (Cuk et al., 2019a,b; Kais et al., 2019) and age (Kais et al., 2019; Cuk et al., 2021), the pacing strategy could also depend on the performance level of the long-distance runners (Nikolaidis and Knechtle, 2017; De Leeuw et al., 2018). Many recent studies examined the influence of performance levels on pacing strategies among long-distance mass-participation races, where the most commonly investigated discipline was marathon (Nikolaidis and Knechtle, 2017; Kais et al., 2019). Only occasional studies had examined other long-distance races, such as 10 km races (Lima-Silva et al., 2010; De Leeuw et al., 2018), half-marathons (De Leeuw et al., 2018), ultramarathons (Knechtle et al., 2022), and running segments in triathlon races (Knechtle et al., 2019a). As a result, many studies using race data with mass participation concluded that runners with a higher level of performance maintained a more even pace compared to runners with lower performance (Santos-Lozano et al., 2014; Nikolaidis and Knechtle, 2017; Kais et al., 2019). The cause for this connection between performance level and even pacing can be found in the amount of carbohydrates (whose quantity is very limited) in metabolic processes increases supralinearly with the running intensity (Romijn et al., 1993). Nevertheless, the most recent study on the “Spartathlon” ultra-marathon showed some contradictions, whereas the fastest and the slowest groups had a more even pace than the two medium groups (Knechtle et al., 2022). Accordingly, we can notice different results depending on the distance examined. Comparing runners’ pacing strategies between the different running disciplines might provide a more robust answer to this issue.

The importance of comparing pacing strategies between the long-distance running races first came from the lack of answers to what mechanism causes a significant decrease in running speed in the second half of the races (i.e., positive pacing strategy). Different authors previously suggested either physiological, sociological, or psychological mechanisms as plausible causes (Roelands et al., 2013; Deaner et al., 2015). However, in recent years, a new methodological approach allowed researchers to provide a more insightful answer to this issue by directly comparing pacing strategies between marathon and half-marathon held on the same event, day and race-track (Cuk et al., 2019a,b). Since the external conditions could impact long-distance pacing strategies, comparison in similar external conditions is important. The results suggest that physiological mechanisms (i.e., glycogen depletion by men, more slow fibers by women) might be the critical factor for the decrease in the running speed later in the race, rather than psychological or sociological reasons (Beltrame et al., 2017). Namely, women had a more even pacing than men in the marathon, but the pacing difference between the sexes almost disappeared in the half-marathon (Cuk et al., 2019a,b) and 10 km (Cuk et al., 2021). This trend was noticed across age groups as well (Cuk et al., 2019a,b). However, no studies have directly compared marathon and half-marathon pacing regarding performance level. Since the previous research on the performance level differences in pacing regarding the individual disciplines showed that differences exist (Hanley, 2014b; Knechtle et al., 2022), it could be significant to examine this factor as well.

Therefore, we aimed to examine the differences in pacing strategies between marathon and half-marathon races regarding their performance levels. We hypothesized that faster runners of both race and sex groups would pace more even than slower runners. We also hypothesized that the half-marathoners of both sexes would show a more even pacing than the marathoners, across all performance groups.

## 2 Materials and methods

### 2.1 Subjects

We initially took into consideration the official results and split times from finishers (*N* = 215,563) in the “Vienna City Marathon” (VCM)^1^ between 2006 and 2018. We then excluded participants who did not complete any of the races, as well as those who did not have a record of any of the segment time. In the end, we considered a total of 208,760 participants for this study. There were 75,492 participants in the marathon (men, *N* = 62,163; women, *N* = 13,329) and 133,268 participants in the half-marathon (men, *N* = 91,145; women, *N* = 42,123). Of the total sample, 153,308 were men, and 55,452 were women. Since the sample size is already large, we choose not to include results after 2019 in the analysis, since the Covid-19 pandemic might indirectly influence performance and pacing strategies (Valenzuela et al., 2021).

The Institutional Review Board of Kanton St. Gallen, Switzerland (Approval number EKSG 01-06-2010) approved this study with a waiver of the requirement for informed consent of the participants as the study involved the analysis of publicly available data. The study was conducted following recognized ethical standards according to the Declaration of Helsinki adopted in 1964 and revised in 2013.

### 2.2 Design

Our study’s experimental approach was observational research.

### 2.3 Methodology

VCM marathon and half-marathon races were held every year on the same day. Both races’ courses were officially certified with a relatively flat track with an elevation difference of only 50 m (ranging from 154 m to 210 m). As a comparison, the Berlin Marathon, considered “the fastest marathon,” has an elevation difference of 21 m. Although VMC is not one of the six major marathons, it has recently been quite popular. Namely, on October 12, 2019, at one-quarter of the VCM course, Eliud Kipchoge ran the marathon distance in 1:59:40.2 h,^2^ becoming the first person to run this distance in less than 2 h, but in specific conditions that aren’t authorized by World Athletics.^3^

The marathon course contained the half-marathon course allowing us to compare these two disciplines. During the race days, the weather temperature was from 7.8°C to 21°C at 9 am and from 10.8°C to 25.1°C at 2 pm. No additional humidity grade or wind speed information was available on the official race website.

### 2.4 Data analysis

#### 2.4.1 Dependent variables

Firstly, we calculated the average running speed in each of the five-race segments and after that, we calculated the average running speed of all five race segments, for each participant in both the marathon and half-marathon (Cuk et al., 2019a,b). Segment 1 corresponds to the initial portion of the races, spanning from the start to the 10th kilometer in the marathon and the 5th km in the half-marathon (first 23.7% of the race). Segment 2 covers the middle portion of the races, ranging from the 10th to the 20th kilometer for the marathon and from the 5th to the 10th kilometer for the half-marathon (23.7–47.4% of the race). Segment 3 represents the latter part of the races, encompassing the 20th to the 30th kilometer for the marathon and the 10th to the 15th kilometer for the half-marathon (47.4–71.1% of the race). Segment 4 corresponds to the final stretch of the races, spanning from the 30th to the 40th kilometer for the marathon and from the 15th to the 20th kilometer for the half-marathon (71.1–94.8% of the race). Segment 5, known as the end spurt (94.8–100% of the race), covers the distance from the 40th kilometer to the finish line for the marathon (42.195 km) and from the 20th kilometer to the finish line for the half-marathon (21.0975 km).

Afterward, we calculated the percentage of the average change of speed for each of the five segments (CS 1–5) concerning the average running speed, whereas all percentages were presented in absolute (i.e., positive) values (Cuk et al., 2019a,b; Knechtle et al., 2022).

Finally, note that one dependent variable depicting pacing is often a better choice since more complex statistics can be performed while pacing on two or more races held on the same track in multiple years can be compared. Therefore, we calculated each participant’s absolute change of speed (ACS) as a mean of the five CSs. This method of data analysis has previously been used (Cuk et al., 2019a,b; Knechtle et al., 2022).

#### 2.4.2 Independent variables

We created four performance level groups (quartiles) concerning the placement in the race (separately for sex and race). Specifically, these groups were established as quartiles within each of four distinct running categories: men marathon runners, men half-marathon runners, women marathon runners, and women half-marathon runners. The High-Level group (HL) consisted of the first quartile of the best-placed runners in each category. Afterward, the Moderate to High-Level group (MHL) consisted of the second-placed quartile in each category. Finally, the Moderate to Low-Level group (MLL) and Low-Level group (LL) consisted of the third and fourth-placed quartiles in each category (Santos-Lozano et al., 2014; Knechtle et al., 2022).

### 2.5 Statistical analyses

Initially, we calculated descriptive statistics of CS and ACS for four performance groups separately for race and sex, as the mean, standard deviation, maximal, and minimal values. Afterward, we confirmed data distribution normality by Kolmogorov–Smirnov, Shapiro–Wilk tests, and visual observation of histograms and q-q plots. Then, we conducted a mixed between-within ANOVA to assess the differences between performance levels on participants’ CS, across five race segments (separately for men and women in half-marathon and marathon). This statistical analysis was used for CS to estimate the main effects of the segment (within-subjects factor), performance level (between-subjects factor), and their interaction (segment × performance level). Finally, we conducted a two-way between-groups ANOVA to assess the differences between performance level and race regarding the ACS (separately for men and women). Accordingly, it was used to assess the main effects of race, performance level, and their interaction. We used the Bonferroni test for all *post hoc* comparisons. The effect size was represented by an eta squared (ŋ^2^) and described using the commonly used guidelines where the values of >0.01, >0.06, and > 0.14 were considered small, moderate, and large effect, respectively, (Cohen, 1988). Since all pacing variables were expressed as percentages, before all ANOVAs, data were log-transformed for the analyses, then back-transformed according to existing methods (Stewart and Hopkins, 2000).

The alpha level was set at *p* < 0.05. We conducted all statistical tests using Microsoft Office Excel 2019 (Microsoft Corporation, Redmond, WA, United States) and IBM SPSS Statistics 26 (IBM, Armonk, NY, United States).

## 3 Results

### 3.1 Pacing strategies

The mean segment speed and the average speed of five segments of all performance groups of men and women are shown in Tables 1, 2, respectively. We observed positive pacing strategies in all men’s and women’s performance groups in both marathon and half-marathon. In addition, most performance groups showed an end spurt, which is noticed as speed acceleration in the last running segment (higher speed in the fifth segment compared to the fourth segment). Notably, faster runners demonstrated less end spurt than slower runners.

**Table 1:** Segments and average speed of five segments for all performance levels of men marathon and half-marathon runners.

Men									
		Marathon (*N* = 62,163)				Half-marathon (*N* = 91,145)			
	DS	LL	MLL	MHL	HL	LL	MLL	MHL	HL
Segment 1 (km/h)	Mean	9.83	10.92	11.87	13.66	9.47	10.63	11.60	13.43
	SD	0.81	0.64	0.66	1.27	0.80	0.62	0.64	1.23
	Min	6.69	7.82	9.28	10.59	5.92	6.82	7.11	10.32
	Max	15.01	15.06	16.39	20.17	16.97	17.27	16.57	21.25
Segment 2 (km/h)	Mean	9.47	10.77	11.76	13.52	9.15	10.46	11.43	13.16
	SD	0.79	0.50	0.52	1.19	0.74	0.42	0.45	1.08
	Min	5.97	8.27	9.26	10.78	5.77	7.11	9.08	10.54
	Max	13.37	15.09	15.61	20.11	14.55	15.27	15.54	20.57
Segment 3 (km/h)	Mean	9.03	10.59	11.66	13.46	8.86	10.38	11.39	13.12
	SD	0.84	0.44	0.45	1.20	0.80	0.41	0.41	1.04
	Min	5.47	7.05	8.29	9.57	5.61	6.23	7.82	8.88
	Max	12.49	13.53	16.14	20.64	12.82	16.01	14.62	20.91
Segment 4 (km/h)	Mean	8.05	9.70	10.81	12.64	8.23	9.89	10.98	12.80
	SD	0.89	0.72	0.70	1.27	0.90	0.64	0.60	1.12
	Min	5.09	6.01	7.17	9.02	5.08	5.44	6.28	8.19
	Max	11.89	12.74	13.74	20.12	11.71	13.13	14.05	20.71
Segment 5 (km/h)	Mean	8.39	9.80	10.81	12.52	8.54	9.99	11.00	12.68
	SD	1.01	0.97	0.96	1.36	1.05	0.87	0.86	1.19
	Min	4.24	4.64	4.91	6.56	4.52	4.67	4.97	5.03
	Max	13.30	14.94	15.02	20.26	14.94	15.00	15.65	19.89
Average speed of 5 segments (km/h)	Mean	8.96	10.36	11.38	13.16	8.85	10.27	11.28	13.04
	SD	0.63	0.34	0.35	1.14	0.67	0.33	0.35	1.03
	Min	6.44	9.45	10.39	11.59	5.69	9.31	10.23	11.56
	Max	10.53	11.41	12.54	20.03	11.39	12.26	12.61	20.58

DS, Descriptive Statistics; Performance groups: LL, Low-Level; MLL, Moderate to Low-Level; MHL, Moderate to High-Level; HL, High-Level.

**Table 2:** Segments and average speed of five segments for all performance levels of women marathon and half-marathon runners.

Women									
		Marathon (*N* = 13,329)				Half-marathon (*N* = 42,123)			
	DS	LL	MLL	MHL	HL	LL	MLL	MHL	HL
Segment 1 (km/h)	Mean	9.07	9.92	10.65	12.10	8.74	9.60	10.35	11.67
	SD	0.61	0.53	0.47	1.29	0.64	0.51	0.50	0.99
	Min	6.72	7.48	9.11	9.10	5.90	6.32	8.00	7.80
	Max	12.26	14.11	12.69	17.70	16.97	11.97	13.79	18.54
Segment 2 (km/h)	Mean	8.62	9.64	10.46	11.95	8.32	9.37	10.17	11.47
	SD	0.58	0.39	10.46	1.22	0.57	0.35	0.34	0.87
	Min	5.98	6.67	9.12	9.90	5.90	8.05	8.09	9.62
	Max	11.22	12.05	12.09	17.94	10.54	11.51	13.96	17.43
Segment 3 (km/h)	Mean	8.35	9.52	10.42	11.95	8.04	9.22	10.09	11.42
	SD	0.61	0.35	0.35	1.19	0.62	0.35	0.34	0.86
	Min	5.70	7.86	8.97	9.88	5.42	6.70	8.22	9.25
	Max	10.30	11.61	11.68	17.96	10.54	11.49	14.25	17.29
Segment 4 (km/h)	Mean	7.75	8.93	9.87	11.41	7.59	8.80	9.71	11.13
	SD	0.68	0.52	0.52	1.18	0.68	0.49	0.48	0.91
	Min	5.10	6.53	7.09	8.13	5.09	6.02	6.77	7.34
	Max	10.06	11.03	11.82	17.68	10.12	11.44	12.07	17.06
Segment 5 (km/h)	Mean	8.17	9.18	10.06	11.48	7.97	9.07	9.86	11.15
	SD	0.79	0.70	0.73	1.21	0.84	0.67	0.67	0.98
	Min	4.51	5.39	5.95	6.33	4.24	4.90	5.50	6.15
	Max	12.33	12.19	13.15	17.80	12.29	12.41	13.58	16.98
Average speed of 5 segments (km/h)	Mean	8.39	9.44	10.29	11.78	8.13	9.21	10.03	11.37
	SD	0.47	0.26	0.28	1.15	0.52	0.25	0.28	0.84
	Min	6.41	8.73	9.54	10.54	5.83	8.34	9.26	10.01
	Max	9.35	10.14	11.08	17.50	9.31	10.09	11.05	17.39

DS, Descriptive Statistics; Performance groups: LL, Low-Level; MLL, Moderate to Low-Level; MHL, Moderate to High-Level; HL, High-Level.

Furthermore, the descriptive data indicated that men marathoners of all performance groups ran faster than men half-marathoners in the first three segments (Table 1).

The results of women (Table 2) indicated that marathoners of all performance levels were faster than half-marathoners throughout all segments of the race.

### 3.2 Performance level × segment (between-within design)

**Figure 1:**
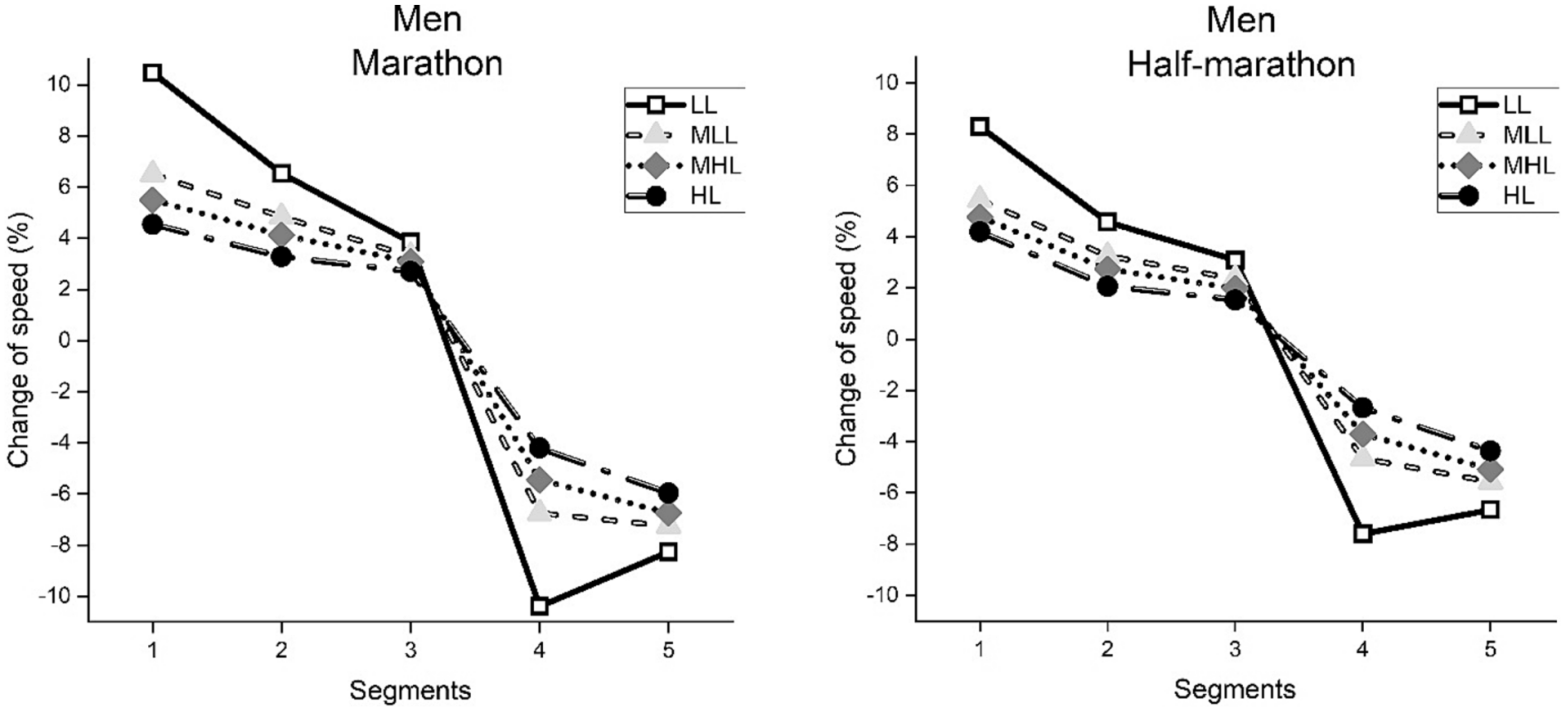
Average speed change in each race segment of men, calculated as a percent change of the mean race speed in marathon and half-marathon. Performance groups: LL, Low-Level; MLL, Moderate to Low-Level; MHL, Moderate to High-Level; HL, High-Level.

**Figure 2:**
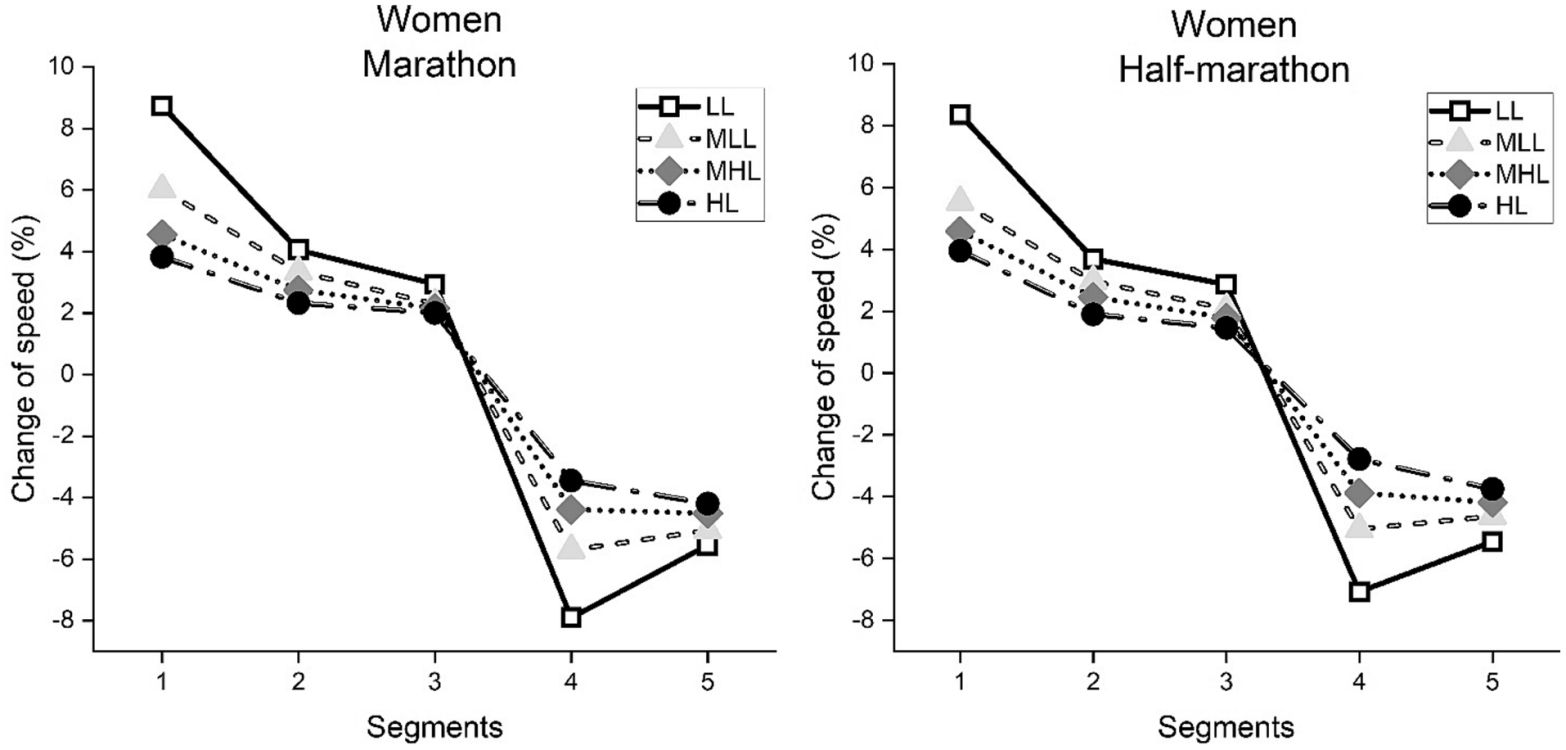
Average change of speed in each race segment of women, calculated as a percent change of the mean race speed in marathon and half-marathon. Performance groups: LL, Low-Level; MLL, Moderate to Low-Level; MHL, Moderate to High-Level; HL, High-Level.

We conducted a mixed between-within analysis to assess the participants’ performance level differences across CS 1–5 (Figures 1, 2). Regarding men marathon runners (Figure 1; left panel), significant main effects for the segment (*F* = 20768.2, *p* < 0.001, ŋ^2^ = 0.691), performance level (*F* = 2957.8, *p* < 0.001, ŋ^2^ = 0.122) and their interaction (*F* = 929.6, *p* < 0.001, ŋ^2^ = 0.187) were observed. Consecutively, in men half-marathon runners (Figure 1; right panel), the same significant main effects for the segment (*F* = 23670.5, *p* < 0.001, ŋ^2^ = 0.779), performance level (*F* = 5343.1, *p* < 0.001, ŋ^2^ = 0.115) and their interaction (*F* = 891.5, *p* < 0.001, ŋ^2^ = 0.106) were observed.

Bonferroni *post hoc* analysis showed significant differences (*p* < 0.001) between CS 1–5 in all performance groups for men marathoners and half-marathoners, indicating a more substantial pacing variability at the beginning and toward the end of the race (i.e., fast start and slow finish, respectfully). Furthermore, significant differences (*p* < 0.001) were observed between performance groups for each CS. Namely, LL showed the greatest variability, while each higher-performance group showed significantly lower variability.

Regarding women marathon runners (Figure 2; left panel), significant main effects for the segment (*F* = 12423.8, *p* < 0.001, ŋ^2^ = 0.813), performance level (*F* = 646.0, *p* < 0.001, ŋ^2^ = 0.094) and their interaction (*F* = 186.8, *p* < 0.001, ŋ^2^ = 0.187) were observed. Consecutively, in women half-marathon runners (Figure 2; right panel), the same significant main effects for the segment (F = 12423.8, *p* < 0.001, ŋ^2^ = 0.866), performance level (*F* = 2891.7, *p* < 0.001, ŋ^2^ = 0.102) and their interaction (*F* = 458.0, *p* < 0.001, ŋ^2^ = 0.134) were observed.

Only in women marathoners in the MHL performance group, Bonferroni’s *post hoc* analysis showed no significant difference between the fourth and fifth segments (*p* > 0.05), indicating no end spurt. In all other performance groups for women marathoners and half-marathoners significant differences (*p* < 0.001) between CS 1–5 were observed, indicating a more substantial pacing variability at the beginning and toward the end of the race (i.e., fast start and slow finish, respectively). Furthermore, significant differences (*p* < 0.001) were observed between performance groups for each CS. Namely, LL showed the greatest variability, while each better-performance group showed significantly lower variability.

### 3.3 Performance level × race (between-between design)

**Figure 3:**
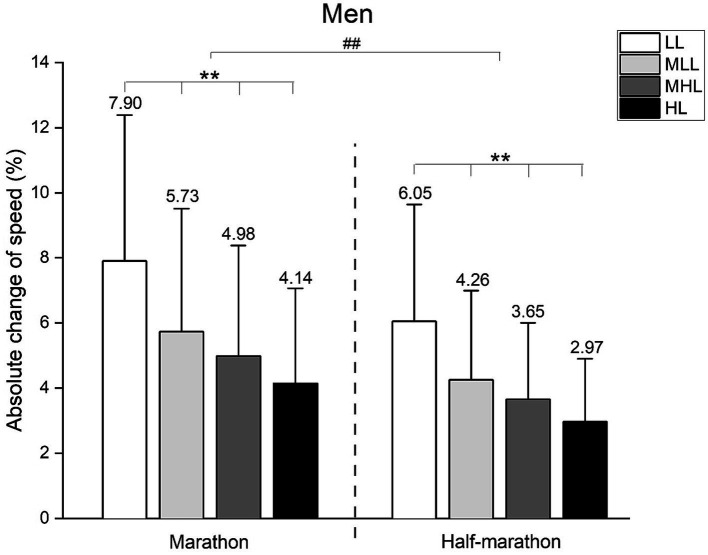
Absolute change of speed in men’s marathon and half-marathon runners. Performance groups: LL, Low-Level; MLL, Moderate to Low-Level; MHL, Moderate to High-Level; HL, High-Level. Error bars represent standard deviation. ** *p* < 0.001; ## *p* < 0.001.

**Figure 4:**
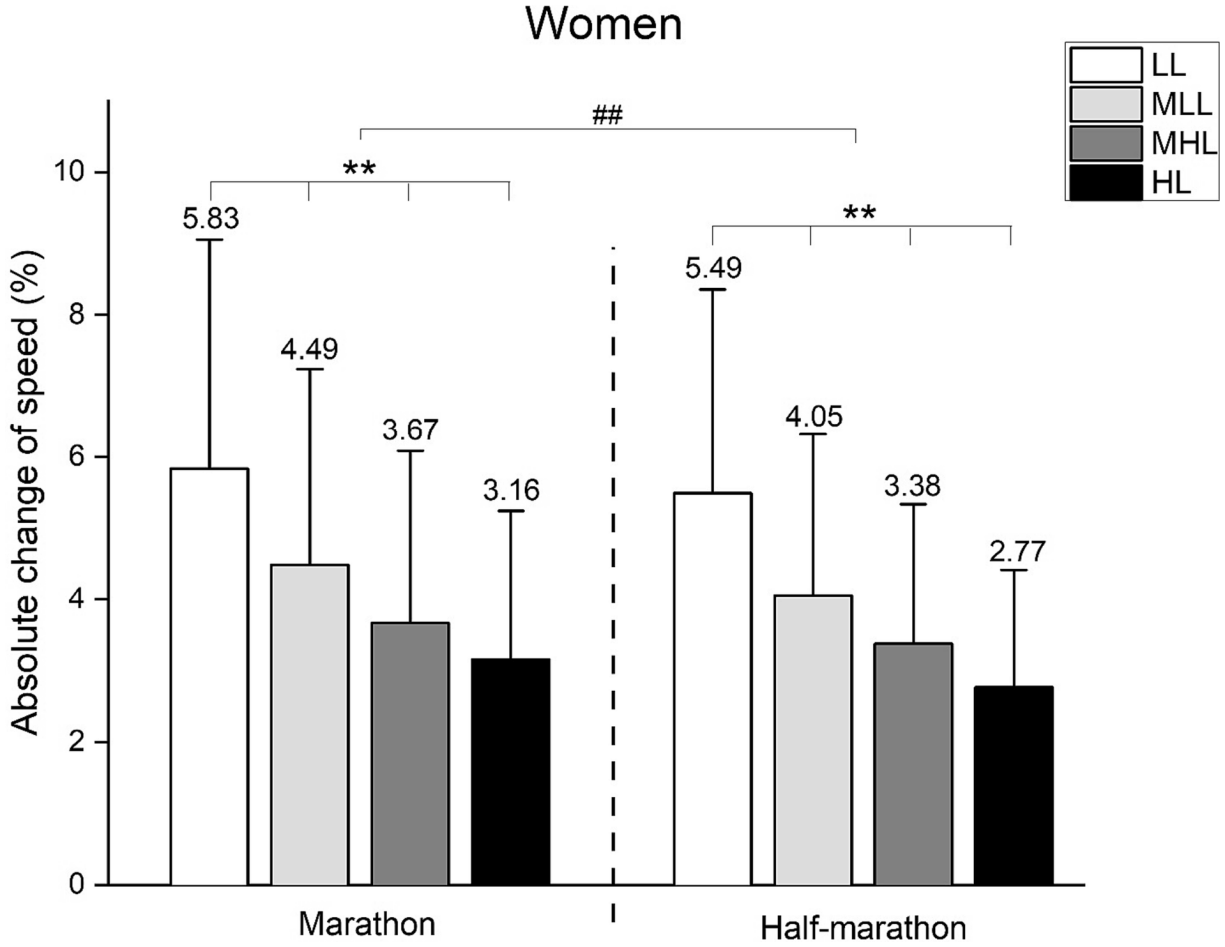
Absolute change of speed in women’s marathon and half-marathon runners. Performance groups: LL, Low-Level; MLL, Moderate to Low-Level; MHL, Moderate to High-Level; HL, High-Level. Error bars represent standard deviation. ** *p* < 0.001; ## *p* < 0.001.

We conducted a two-way between-groups ANOVA to assess the differences between performance level and race regarding the ACS, separately for men and women (Figures 3, 4, respectively).

Regarding men (Figure 3), significant main effects for the race (*F* = 7896.0, *p* < 0.001, ŋ^2^ = 0.246), and performance level (*F* = 7980.2, *p* < 0.001, ŋ^2^ = 0.746) were observed, but with the trivial practical significance of their interaction (*F* = 80.1, *p* < 0.001, ŋ^2^ = 0.007). Bonferroni *post hoc* analysis was presented in Figure 3 (** indicating a significant difference between performance levels at *p* < 0.01; ## indicating a significant difference between races at *p* < 0.01).

Furthermore, in women (Figure 4), the same significant main effects for the race (*F* = 247.7, *p* < 0.001, ŋ^2^ = 0.032) and performance level (*F* = 2527.5, *p* < 0.001, ŋ^2^ = 0.968) were observed. Conversely, race and performance level interaction showed no significance (*F* = 1.7, *p* = 0.168, ŋ^2^ < 0.001). Bonferroni *post hoc* analysis was presented in Figure 4 (** indicating a significant difference between performance levels at *p* < 0.01; ## indicating a significant difference between races at *p* < 0.01).

## 4 Discussion

The main aim of our study was to assess and compare pacing strategies between marathon and half-marathon runners regarding their performance levels. We confirmed the hypothesis that faster runners of both race and sex would pace more evenly (i.e., presenting smaller changes in speed) than slower runners. Also, we partially confirmed the hypothesis that the half-marathoners of both sexes would show more even pacing than the marathoners across all performance groups, since the difference between ACS of women in marathon and half-marathon has a trivial effect size.

### 4.1 Pacing strategies

We observed positive pacing strategies in all men’s and women’s performance groups in both marathon and half-marathon. The most significant speed decline was observed in the 4th segment, which is more pronounced in the slower groups. In addition, most performance groups showed an end spurt. Notably, faster runners demonstrated less end spurt than slower runners, which was consistent with the smaller changes in speed between segments.

The most significant speed decline in the 4th segment could be explained by a large amount of fatigue accumulated due to the fast start and the long distance covered (Hanley, 2014a; Cuk et al., 2019a,b).

Intriguingly, both men and women were faster in the marathon than in the half-marathon. Since there were 57% more participants in the half-marathon than in the marathon race (133,268 vs. 75,492), this can be explained by the fact that the increase in the number of runners can worsen the results (Vitti et al., 2020). We can assume that a larger proportion of participants in the half-marathon, compared to the marathon, consists of recreational runners. Notably, in women, the half-marathon-marathon ratio is approximately 3:1, while in men, this ratio is close to 2:1. Furthermore, endurance performance can be affected by previous running experience (Chow et al., 2011). Since more runners participate in the half-marathon than the marathon, as it is more available distance, we can assume that many recreational runners and beginners (i.e., inexperienced runners) decreased the mean running speed.

### 4.2 Performance level × segment (between-within design)

The results of this study show a significant impact of performance level on participants’ running variability across five race segments in both race and sex groups.

Overall, the results of our study indicate that higher-performance runners showed less pace variability than runners with lower performance in both race and sex groups. A similar conclusion was reached in other studies that examined this topic in only marathon races (Nikolaidis and Knechtle, 2017; Kais et al., 2019). It is also interesting that a more significant pace decline during the half-marathon was observed in runners who ran slower than the predicted finishing time, while those who ran faster than the predicted finishing time had an even pace (Piacentini et al., 2019). So, a steady pace could be the most metabolically efficient strategy for finishing a long-distance race (Rapoport, 2010). If we relate this with our results, it can be assumed that the higher-performance runners achieved even better results than expected due to the steady pacing. Namely, runners with a more prominent change of running speed have higher energy expenditure and, consequently, poorer performance.

All women groups showed an end spurt, but it was more pronounced in lower-performance groups. However, there is no end spurt in the HL and MHL performance groups of men in the marathon, while in the half-marathon, the end spurt is absent only in the HL performance group. The missing end spurt in high-performance runners can be explained by better energy distribution, due to their accurate self-assessment and inability to make sudden changes at the end. It could indicate that physiological factors are more pronounced compared to psychological factors. In contrast, since pacing involves anticipation, awareness of the end-point, experience, and sensory feedback (Skorski and Abbiss, 2017), knowing the proximity of the finish line can motivate runners to utilize their final reserves, indicating psychological factors rather than physiological ones. Accordingly, the pronounced end spurt reflects the runners’ greater caution and energy reserve during the race to avoid facing complete exhaustion before the end (de Koning et al., 2011). Because of that reserve, they also have more energy for the end of the race, which is characteristic of the slower marathon (Nikolaidis and Knechtle, 2017) and half-marathon (Hanley, 2014a) runners.

Several factors have been shown to affect the pacing. These factors are physiological, biomechanical, and psychological. Within them, thermoregulation, reduction of glycogen stores, neuromuscular fatigue, and increased rate of perceived exertion (RPE) have a direct impact (Foster et al., 2023). In particular, the amount of carbohydrates in metabolic processes increases supralinearly with the running intensity (Romijn et al., 1993). For example, if the running speed is gradually decreasing and then increasing over time to achieve the target result, in the compensatory fast running part, carbohydrate utilization exceeds the utilization amount it would have been had the runner maintained his target pace. Additionally, carbohydrate utilization during the fast part is also greater in size than the carbohydrate savings achieved during the slow interval. As a result, the net carbohydrate utilization is higher than if the runner never varied from the target pace (Rapoport, 2010). Increased glycogen depletion is a potential cause of decreased intensity (Abbiss and Laursen, 2008). This has resulted in altered substrate use, neuromuscular fatigue, and/or psychological factors associated with fatigue perception (Abbiss and Laursen, 2008). Some authors suggest that speed decline can be explained with the Hazard Score that indicates homeostatic disturbance computed as a product of momentary RPE with the fraction of the remaining race distance (de Koning et al., 2011; Foster et al., 2023).

Furthermore, climatic conditions and outside temperature can affect pace and performance during a long-distance race (Trubee et al., 2014), while slower marathoners and novices had less knowledge about the type and importance of hydration (Namineni et al., 2021). Since the VCM race started every year in the early morning hours, with the temperature fresher than in the later hours (as mentioned in the methodology section), the slower runners finished the race always in the warmer part of the day compared to the faster ones. Considering that an increased outside temperature of 1°C can decrease performances (Knechtle et al., 2019b), a significant pace decline during the race in slower runners could also be caused by the outside temperature rise.

Knowledge of the race endpoint is considered the most important factor according to which the strategy of running pace is established (St Clair Gibson et al., 2006; de Koning et al., 2011). The long-distance pacing profile is reflected in the control of physiological processes by changes in muscle activation in an anticipatory manner (de Koning et al., 2011; Foster et al., 2023). Namely, the pacing profile is formed based on physiological systems and experience feedback. It takes experience to gain the necessary knowledge of environmental conditions, internal metabolic functions, and fuel reserves for a given race distance (St Clair Gibson et al., 2006; Foster et al., 2023). Also, since we know that physiological adaptation processes require a certain period to occur, a lower level of performance is expected in less running experience groups (Granata et al., 2018). Thus, the number of years of running and previous races are directly related to the race pace, regardless of age, gender, and current result. Therefore, experienced runners are expected to have a more even pace, while less experienced ones have a more significant decline (Deaner et al., 2015), which agrees with the result of our research.

### 4.3 Performance level × race (between-between design)

A significant impact of performance level and race on ACS in both men and women was observed. However, the difference between ACS of women in half-marathon and marathon has a trivial effect size.

In all performance groups, men marathon runners had more pacing variability than half-marathon runners. Interestingly, the HL group in the marathon has similar variability to the MLL in the half-marathon. The LL group of men marathon runners showed the greatest variability of pace, including sex and race. According to the previous assumptions, one of the factors influencing this pace variability of slower runners may be the increasing outside temperature as the race progresses (Trubee et al., 2014), which could increase the heat stress of these runners and decrease the speed as well (Leslie et al., 2023). A similar reason can be assumed as the cause of greater variability in the pace of marathoners compared to half-marathoners. Half-marathoners, as well as faster runners, spend less time on the track while the temperature is still lower.

Regarding women, there are significant differences in pace variability between marathon and half-marathon, but it is less pronounced and has little practical significance (ŋ^2^ = 0.032). A higher relative number of slow-twitch muscle fibers in women could be one of the reasons for faster dynamics of oxygen extraction in peripheral and pulmonary blood at moderate exercise intensities compared to men (Beltrame et al., 2017). In general, muscle metabolism in women is more capable of synthesizing ATP from oxidative phosphorylation (Ansdell et al., 2020). Additionally, there is some evidence that women are more likely to have a better strategy that reduces the risk of developing heat illness on exertion (Périard et al., 2017). These could be the main reasons for the less pronounced differences in pace variability between half-marathon and marathon, caused by women’s physiology. However, there is certainly a very similar difference between performance groups for men.

In summary, the results of this study show a significant impact of performance level on participants’ pace variability across the race in both race and sex groups. Namely, runners of a higher level of performance showed more even pacing than runners of a lower level of performance. Also, there was a significant impact of race format on ACS in both men and women. However, the difference between ACS of women in marathon and half-marathon has a trivial effect size.

### 4.4 Practical applications

Given the growing number of runners worldwide at these distances, and the common practice to participate in half-marathon and marathon races interchangeably, the results of this study are of great practical value. A large percentage of the participants in the VCM^1^ were foreign runners, so the influence of only one nation can be ruled out. Given the vast sample of participants, which probably well describes the population of runners, the results can be generalized. Since we know about excellent consistency of the pacing profile in the same race from edition to edition, it is possible to plan the pacing strategy. Namely, coaches and runners could adjust their training programs and race pacing strategy concerning the level of performance of each runner to achieve the best possible result and reduce the risk of over-exhaustion. A more specific practical implication of these results suggests that slower runners in both races, but especially men in marathon distance, should start the race at a slower pace, aiming to maintain a more even pace. Therefore, with this group of runners, compared to all others, a significant emphasis should be placed on training to practice running at an even pace. By doing so, they can efficiently utilize their energy and distribute it more effectively from the beginning of the race. Otherwise, there is a significant risk of depleting their energy early or excessively conserving it, which would likely lead to compensating through an “end spurt,” as well as worsening performance.

## 5 Limitation of this study

A limitation of this study is the lack of additional information on other factors that could affect the pacing, such as previous training routine, experience in running and racing, or anthropometric characteristics. Considering that performance groups of runners have been created separately for sexes and races during the 13 years of the VCM, different weather conditions and number of participants in different years may have an impact on the consistency of the divisions. Further research on pacing in the marathon and half-marathon is desirable, including additional information on a previous training routine, motivation, running and racing experience, or anthropometric characteristics.

## Data availability statement

The raw data supporting the conclusions of this article are available online on the official website of the Vienna City Marathon, https://www.vienna-marathon.com/?go=result.

## Ethics statement

The Institutional Review Board of Kanton St. Gallen, Switzerland (Approval number EKSG 01-06- 2010) approved this study with a waiver of the requirement for informed consent of the participants as the study involved the analysis of publicly available data.

## Author contributions

LR: Conceptualization, Writing – original draft. IC: Writing – original draft, Formal analysis. EV: Data curation, Writing – review & editing. SS: Writing – review & editing, Conceptualization. PN: Investigation, Writing – review & editing. KW: Writing – review & editing. BK: Writing – review & editing, Supervision.

## Footnotes

^1^Vienna City Marathon. (2022). https://www.vienna-marathon.com [Accessed March 10, 2022].

^2^INEOS 1:59 Challenge. (2022). https://www.ineos159challenge.com/ [Accessed March 10, 2022].

^3^World Athletics (2023). Available at: https://worldathletics.org/competitions. [Accessed September 13, 2023].

## References

[ref1] Abbiss C. R. Laursen P. B. (2008). Describing and understanding pacing strategies during athletic competition. Sports Med. 38, 239–252. doi: 10.2165/00007256-200838030-00004 18278984

[ref2] Ansdell P. Thomas K. Hicks K. M. Hunter S. K. Howatson G. Goodall S. (2020). Physiological sex differences affect the integrative response to exercise: acute and chronic implications. Exp. Physiol. 105, 2007–2021. doi: 10.1113/EP088548 33002256

[ref3] Beltrame T. Rodrigo V. Hughson R. L. (2017). Sex differences in the oxygen delivery, extraction, and uptake during moderate-walking exercise transition. Appl. Physiol. Nutr. Metab. 42, 994–1000. doi: 10.1139/apnm-2017-0097 28570840

[ref4] Chow Voight A. M. Roberts B. Lunos S. (2011). Pre- and postmarathon training habits of nonelite runners. Open Access J. Sports Med. 2, 13–18. doi: 10.2147/OAJSM.S16665, PMID: 24198565 PMC3781877

[ref5] Cohen J. (1988) Statistical power analysis for the behavioral sciences. 2nd. Lawrence Erlbaum Associates: Hillsdale, NJ, USA.

[ref6] Cuk I. Nikolaidis P. T. Knechtle B. (2019a). Sex differences in pacing during half-marathon and marathon race. Res. Sports Med. 28, 111–120. doi: 10.1080/15438627.2019.1593835, PMID: 30897961

[ref7] Cuk I. Nikolaidis P. Markovic S. Knechtle B. (2019b). Age differences in pacing in endurance running: comparison between Marathon and half-Marathon men and women. Medicina 55:479. doi: 10.3390/MEDICINA55080479, PMID: 31416198 PMC6723688

[ref8] Cuk I. Nikolaidis P. T. Villiger E. Knechtle B. (2021). Pacing in long-distance running: sex and age differences in 10-km race and Marathon. Medicina 57:389. doi: 10.3390/medicina 33920504 PMC8073231

[ref9] de Koning J. J. Foster C. Bakkum A. Kloppenburg S. Thiel C. Joseph T.. (2011). Regulation of pacing strategy during athletic competition. PLoS One 6:e15863. doi: 10.1371/JOURNAL.PONE.0015863, PMID: 21283744 PMC3024328

[ref10] De Leeuw A. W. Meerhoff L. A. Knobbe A. (2018). Effects of pacing properties on performance in long-distance running. Big Data 6, 248–261. doi: 10.1089/big.2018.0070, PMID: 30421990

[ref11] Deaner R. O. Carter R. E. Joyner M. J. Hunter S. K. (2015). Men are more likely than women to slow in the Marathon. Med. Sci. Sports Exerc. 47, 607–616. doi: 10.1249/MSS.0000000000000432, PMID: 24983344 PMC4289124

[ref12] Foster C. de Koning J. J. Hettinga F. J. Barroso R. Boullosa D. Casado A.. (2023). Competition between desired competitive result, tolerable homeostatic disturbance, and psychophysiological interpretation determines pacing strategy. Int. J. Sports Physiol. Perform. 18, 335–346. doi: 10.1123/IJSPP.2022-0171 36848906

[ref13] Granata C. Jamnick N. A. Bishop D. J. (2018). Principles of exercise prescription, and how they influence exercise-induced changes of transcription factors and other regulators of mitochondrial biogenesis. Sports Med. 48, 1541–1559. doi: 10.1007/S40279-018-0894-4, PMID: 29675670

[ref14] Hanley B. (2014a). Pacing profiles and pack running at the IAAF world half Marathon championships. J. Sports Sci. 33, 1189–1195. doi: 10.1080/02640414.2014.988742, PMID: 25483017

[ref15] Hanley B. (2014b). Senior men’s pacing profiles at the IAAF world cross country championships. J. Sports Sci. 32, 1060–1065. doi: 10.1080/02640414.2013.878807 24506144

[ref16] Kais Ü. Pind R. Pehme A. Kaasik P. Mooses M. (2019). Pacing strategy of the finishers of the world marathon majors series. Kinesiology 51, 22–27. doi: 10.26582/k.51.1.5

[ref17] Knechtle B. Cuk I. Villiger E. Nikolaidis P. T. Weiss K. Scheer V.. (2022). The effects of sex, age and performance level on pacing in ultra-Marathon runners in the “Spartathlon”. Sports Med. 8, 69–10. doi: 10.1186/S40798-022-00452-9, PMID: 35552909 PMC9106765

[ref18] Knechtle B. di Gangi S. Rüst C. A. Villiger E. Rosemann T. Nikolaidis P. T. (2019b). The role of weather conditions on running performance in the Boston Marathon from 1972 to 2018. PLoS One 14:e0212797. doi: 10.1371/JOURNAL.PONE.0212797, PMID: 30849085 PMC6407773

[ref19] Knechtle B. Käch I. Rosemann T. Nikolaidis P. T. (2019a). The effect of sex, age and performance level on pacing of ironman triathletes. Res. Sports Med. 27, 99–111. doi: 10.1080/15438627.2018.1546703 30418036

[ref20] Leslie E. Ducharme J. B. Coffey P. van Horn M. L. (2023). Pacing and heat stress independently and differentially effect elite marathon performance. Sport Sciences for Health 19, 359–367. doi: 10.1007/s11332-022-01034-8

[ref21] Lima-Silva A. E. Bertuzzi R. C. M. Pires F. O. Barros R. V. Gagliardi J. F. Hammond J.. (2010). Effect of performance level on pacing strategy during a 10-km running race. Eur. J. Appl. Physiol. 108, 1045–1053. doi: 10.1007/S00421-009-1300-6 20012450

[ref22] Namineni N. Potok O. A. Ix J. H. Ginsberg C. Negoianu D. Rifkin D. E.. (2021). Marathon runners’ knowledge and strategies for hydration’. Clin. J. Sport Med. 32, 517–522. doi: 10.1097/JSM.0000000000000990, PMID: 34723866 PMC9050964

[ref23] Nikolaidis P. T. Knechtle B. (2017). Effect of age and performance on pacing of marathon runners. J. Sports Med. 8, 171–180. doi: 10.2147/oajsm.s141649, PMID: 28860876 PMC5571841

[ref24] Périard J. D. Racinais S. Timpka T. Dahlström Ö. Spreco A. Jacobsson J.. (2017). Strategies and factors associated with preparing for competing in the heat: a cohort study at the 2015 IAAF world athletics championships. Br. J. Sports Med. 51, 264–270. doi: 10.1136/BJSPORTS-2016-096579, PMID: 27815238 PMC5318647

[ref25] Piacentini M. F. Reda D. Minganti C. Baldassarre R. Tarperi C. Festa L.. (2019). Pacing profiles of master athletes according to their predicted finishing time. Move. Sports Sci. 2, 37–44. doi: 10.1051/SM/2019016

[ref26] Rapoport B. I. (2010). Metabolic factors limiting performance in Marathon runners. PLoS Comput. Biol. 6:e1000960. doi: 10.1371/JOURNAL.PCBI.1000960, PMID: 20975938 PMC2958805

[ref27] Roelands B. de Koning J. Foster C. Hettinga F. Meeusen R. (2013). Neurophysiological determinants of theoretical concepts and mechanisms involved in pacing. Sports Med. 43, 301–311. doi: 10.1007/S40279-013-0030-4, PMID: 23456493

[ref28] Romijn J. A.. (1993). Regulation of endogenous fat and carbohydrate metabolism in relation to exercise intensity and duration. Am. J. Phys. 265, E380–E391. 10.1152/ajpendo.1993.265.3.E380 8214047

[ref29] Santos-Lozano A. Collado P. Foster C. Lucia A. Garatachea N. (2014). Influence of sex and level on Marathon pacing strategy. Insights from the new York City race. Int. J. Sports Med. 35, 933–938. doi: 10.1055/S-0034-1367048 24886929

[ref30] Skorski S. Abbiss C. R. (2017). The manipulation of pace within endurance sport. Front. Physiol. 8:102. doi: 10.3389/FPHYS.2017.00102 28289392 PMC5326767

[ref31] St Clair Gibson A. Lambert E. V. Rauch L. H. G. Tucker R. Baden D. A. Foster C.. (2006). The role of information processing between the brain and peripheral physiological Systems in Pacing and Perception of effort. Sports Med. 36, 705–722. doi: 10.2165/00007256-200636080-00006, PMID: 16869711

[ref32] Stewart A. M. Hopkins W. G. (2000). Seasonal training and performance of competitive swimmers. J. Sports Sci. 18, 873–884. doi: 10.1080/026404100750017805 11144864

[ref33] Trubee N. W. Vanderburgh P. M. Diestelkamp W. S. Jackson K. J. (2014). Effects of heat stress and sex on pacing in marathon runners. J. Strength Cond. Res. 28, 1673–1678. doi: 10.1519/JSC.0000000000000295 24149746

[ref34] Tucker R. Noakes T. D. (2009). The physiological regulation of pacing strategy during exercise: a critical review. Br. J. Sports Med. 43:e1. doi: 10.1136/BJSM.2009.057562, PMID: 19224909

[ref35] Valenzuela P. L. Rivas F. Sánchez-Martínez G. (2021). Effects of COVID-19 lockdown and a subsequent retraining period on elite athletes workload, performance, and autonomic responses: a case series. Int. J. Sports Physiol. Perform. 16, 1707–1711. doi: 10.1123/IJSPP.2020-0735 33873155

[ref36] Vitti A. Nikolaidis P. T. Villiger E. Onywera V. Knechtle B. (2020). The “new York City Marathon”: participation and performance trends of 1.2M runners during half-century. Res. Sports Med. 28, 121–137. doi: 10.1080/15438627.2019.1586705, PMID: 30889965

